# Impact of the Emergency Procedure Zone on Emergency Care

**DOI:** 10.3390/medicina59050901

**Published:** 2023-05-08

**Authors:** I-Chen Lin, Po-Wei Chiu, Chih-Hao Lin

**Affiliations:** Department of Emergency Medicine, College of Medicine, National Cheng Kung University Hospital, National Cheng Kung University, Tainan 70403, Taiwan

**Keywords:** emergency department, design, workflow, procedure, ultrasound

## Abstract

*Background:* Emergency department (ED) overcrowding is a public health crisis that affects patient care quality. Space management in the ED can affect patient flow dynamics and clinical practice. We proposed a novel design of the “emergency procedure zone” (EPZ). The purpose of the EPZ was to provide an isolated area for clinical practice and procedure teaching, to ensure a secure area with adequate equipment and monitors, and safeguard patient privacy and safety. This study aimed to analyze the impact of the EPZ on procedural practice and patient flow dynamics. *Methods:* This study was conducted at the ED of a tertiary teaching hospital in Taiwan. Data were collected from 1 March 2019 to 31 August 2020 (pre-EPZ period) and from 1 November 2020 to 30 April 2022 (post-EPZ period). Statistical analyses were performed using IBM SPSS Statistics software. This study focused on the number of procedures and length of stay in the emergency department (LOS-ED). Variables were analyzed using the chi-square test and Mann–Whitney U test. Statistical significance was defined as *p* < 0.05. *Results:* There were 137,141 (pre-EPZ period) and 118,386 (post-EPZ period) ED visits recorded during this period. The post-EPZ period showed a significant increase in central venous catheter insertion, chest tube or pigtail placement, arthrocentesis, lumbar puncture, and incision and drainage procedures (*p* < 0.001). For patients who were directly discharged from the ED, the post-EPZ period also had a higher percentage of ultrasound studies performed in the ED and a shorter LOS-ED for patients who were directly discharged from the ED (*p* < 0.001). *Conclusions:* The establishment of an EPZ in the ED has a positive impact on procedural efficiency. The EPZ improved diagnosis and disposition efficiency, shortened the length of stay, and provided benefits such as improved management, patient privacy, and teaching opportunities.

## 1. Introduction

Emergency department (ED) overcrowding affects the quality of patient care and is becoming a public crisis [1,2]. ED overcrowding can be attributed to three components of patient flow: input, throughput, and output [3,4]. Each component is multifactorial [4]. The most straightforward way to address ED crowding is to decrease the demand for inputs and increase the throughput and output capacity [5,6]. Decreasing input is often limited by the capacity of neighboring hospitals, changes in the prehospital ambulance system, or adjustments in the overall culture of care. To increase the output capacity, the hospital’s admission process must be optimized and bed capacity should be increased [7,8]. The medical team in the ED can increase the throughput capacity by re-engineering the emergency operation lines and medical flow of the ED, remodeling the environment, re-evaluating the patient–caregiver ratio, and creating a new model of medical care [9,10]. These approaches provide opportunities to increase throughput capacity and achieve qualitative changes in the medical experience by redesigning the physical environment to ensure a safer and more efficient flow of operations in the ED.

Separating patients into different zones according to their clinical needs is a new concept that improves patient flow efficiency in the ED [11]. Conversely, setting a clinical procedure zone is not a unique or novel concept; many EDs are designed with such a space, often known as a “procedure room”. Most EDs have a separate space for surgical intervention and patients are transferred to the resuscitation bay for physiological monitoring when procedural sedation is required [12]. There is no requirement for a separate group of medical personnel to maintain procedural quality and process control or manage instruments or physiological monitors.

Space management in the ED can affect patient flow dynamics and clinical practice. Due to space limitations, ED physicians may need to perform clinical procedures directly at the bedside, including diagnostic ultrasound imaging and invasive treatments [13]. However, the layout of most EDs, especially with overcrowding, makes it difficult to ensure patient safety and privacy. Patients require different physiological monitoring modules during treatment, depending on the severity of disease. However, clinical procedures can be delayed due to the inadequacy of space or unavailability of equipment. The postponement of necessary intervention and treatment could jeopardize patient outcomes and exacerbate ED overcrowding.

To cope with space limitations, we propose a novel “emergency procedure zone” (EPZ) design. The purpose of the EPZ was to provide an isolated area for clinical practice and procedure teaching, ensure a secure area with adequate equipment and monitors, and safeguard patient privacy and safety. We conducted a study to analyze the impact of the EPZ on procedural practice and patient flow.

## 2. Methods

### 2.1. Emergency Department

A retrospective cohort study was conducted in the Emergency Department of National Cheng Kung University Hospital, a tertiary teaching hospital in Taiwan. The ED has 34 attending physicians and 13 residents, and the annual number of patient visits is approximately 85,000. The ED has several clinical zones to manage different types of patients, including ambulatory emergency care, acute care, resuscitation, and critical care.

### 2.2. Emergency Procedure Zone

Prior to the establishment of the EPZ, most clinical examinations and procedures were performed at the patient’s bedside and the equipment was obtained from the equipment cabinet or a storage area. Due to space and resource limitations, sonographic imaging was regularly performed in sonography rooms by radiologists or cardiologists, but not in the ED. The EPZ was established in September 2020 and involved adding an additional team of personnel beyond the existing staff, with each shift consisting of one nurse, one ED resident, and one ED attending physician.

**Figure 1:**
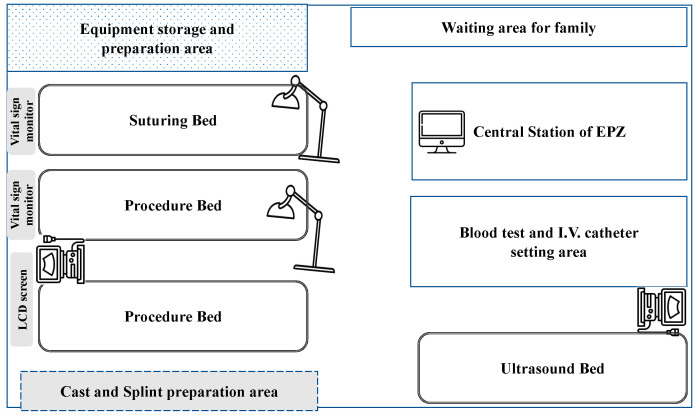
Layout of emergency procedure zone in the emergency department.

The EPZ is isolated from the triage area, waiting area, consulting room, resuscitation room, treatment zone, and observation zone. The space was approximately 52 m^2^ and contained four areas: administration, preparation, waiting, and treatment. The layout of the EPZ is presented in Figure 1.

The treatment area was multifunctional and equipped with one ultrasound bed, one suturing bed, and two procedure beds. An ultrasound bed was used for point-of-care ultrasonography (POCUS) and other diagnostic sonographic imaging procedures. A suturing bed was used for wound management, incision and drainage, and foreign body removal. The procedure beds were used for various procedures, including central venous catheter (CVC) insertion, thoracocentesis, abdominocentesis, placement of chest tubes or pigtails, arthrocentesis, nerve blocks, lumbar punctures, reduction of dislocation, and splint/casting for bone fractures.

If a patient required a diagnostic survey or clinical intervention, physicians in the clinical zones would input the indication electronically and submit a request. Following submission, the resident in the EPZ first reviewed the request and confirmed that the patient met the indications for the procedure, with contraindications excluded. The EPZ nurse assisted in arranging and scheduling the procedure, preparing equipment, moving the patient to the EPZ, and applying appropriate physiological monitoring. The resident then performed the clinical procedures under the supervision of an attending emergency physician. When the procedure was complete, the resident documented the examination findings and procedural notes. For critically ill patients, the procedures were performed at the patient’s bedside and not in the EPZ.

### 2.3. Data Collection

The EPZ was established in September 2020. The data prior to EPZ establishment were collected from 1 March 2019 to 31 August 2020 (pre-EPZ period), and the data subsequent to EPZ establishment were collected from 1 November 2020 to 30 April 2022 (post-EPZ period). Due to the integration of the administrative system, a buffer time of 2 months was used in this study.

The data were collected via an emergency medical computerized system, which included patients’ basic characteristics such as sex or age; triage information such as acuity levels or reasons for visits; final disposition; and length of stay in the ED (LOS-ED). The EPZ clinical procedures evaluated in this study included sutures, CVC insertion, placement of chest tubes or pigtails, thoracocentesis, abdominocentesis, arthrocentesis, lumbar punctures, incision and drainage, reduction of dislocation, splinting, POCUS performed by ED physicians, and sonographic imaging performed by radiologists or cardiologists.

### 2.4. Data Analysis

We analyzed the number of procedures performed in the pre- and post-EPZ periods as well as the LOS-ED using multivariate analysis tests. LOS-ED is multifactorial and largely depends on the output domain, such as the availability of hospital beds in wards and intensive care units. Therefore, we focused only on patients who were directly discharged from the ED, which was greatly influenced by the availability and efficiency of clinical examinations or procedures.

Statistical analyses were performed using the IBM SPSS Statistics software (version 25.0; SPSS Inc., Chicago, IL, USA). The chi-square test was used for categorical variables and the Mann–Whitney U test was used for continuous variables. Statistical significance was defined as *p* < 0.05.

## 3. Results

A total of 137,141 (pre-EPZ) and 118,386 (post-EPZ) ED visits were recorded during the study period. Patient data are presented in Table 1.

Table 2 describes the clinical procedures performed during the pre- and post-EPZ periods. The post-EPZ period had a significantly higher (*p* < 0.001) incidence of CVC insertion (pre-EZP = 0.45%; post-EZP = 1.33%), chest tube or pigtail placement (pre-EZP = 0.21%; post-EZP = 0.29%), arthrocentesis (pre-EZP = 0.03%; post-EZP = 0.11%), lumbar puncture (pre-EZP = 0.11%; post-EZP = 0.34%), and incision and drainage (pre-EZP = 0.02%; post-EZP = 0.10%) in comparison to the pre-EPZ period. The post-EPZ period also recorded significantly more ultrasound (pre-EPZ = 7.34%; post-EPZ = 10.18%; *p* < 0.001) and ultrasound scans performed in the ED (pre-EPZ = 1.48%; post-EPZ = 4.54%; *p* < 0.001) as well as fewer ultrasound scans performed in the radiology/cardiology department (pre-EPZ = 5.86%; post-EPZ = 5.65%; *p* = 0.022).

For those who were directly discharged from the ED (Table 3), the post-EPZ period also had significantly more ultrasound and ultrasound scans performed in the ED (*p* < 0.001) and fewer ultrasound scans performed in the radiology or cardiology departments (*p* < 0.001).

The LOS-ED scores of the patients who were directly discharged from the ED are shown in Table 4. The patients during the post-EPZ period had significantly shorter LOS-ED (pre-EPZ = 110 min; post-EPZ = 102 min; *p* < 0.001).

## 4. Discussion

The establishment of an EPZ affects the number and types of procedures performed in the ED. Compared with the pre-EPZ period, the post-EPZ period observed a decrease in the mean total number of ED visits per day, with a decrease in the proportion of trauma patients. This may have been due to a reduction in unnecessary outings during the COVID-19 pandemic [14]. However, the total number of procedures performed per month, including CVC, chest tube, pigtail, lumbar puncture, and bedside incision and drainage, increased significantly compared to that before the establishment of the EPZ. Further, the incidence of lumbar puncture also increased significantly, suggesting that ED physicians may have previously delayed performing these procedures. The number of “suture” procedures performed decreased, which was likely due to reduced incidences of trauma during the pandemic.

The number of POCUS procedures performed by ED physicians, including both trauma and diagnostic ultrasound imaging, significantly increased during the post-EPZ period. This suggests that prior to the establishment of the EPZ, POCUS was infrequently used in the ED, and emergency physicians had to rely on the radiology department for ultrasound examinations. POCUS is currently regarded as an important tool to aid in rapid patient diagnosis [15]. Increasing POCUS use in the ED positively affects the efficiency of diagnosis and disposition and, as a result, shortens the LOS-ED [16,17]. This was also reflected in our findings, which suggest that the establishment of an EPZ may improve efficiency in the ED, particularly with respect to POCUS, procedures performed, and LOS-ED. However, we also need to consider that during the post-EPZ period, there was a decrease in the daily patient volume (Table 2). This may also be one of the factors affecting the LOS-ED.

Table 4 indicates a reduction in the median LOS-ED for all patients, with some procedures showing an increase in LOS post-EPZ implementation. To explain this, during the pre-EPZ period, certain procedures were performed in the emergency department with the assistance of specialists, while in the post-EPZ period, ED residents or attending physicians performed all procedures. Consequently, the procedure process may have been slower. However, the widespread use of POCUS expedited the diagnostic process, resulting in the observed decrease in median LOS-ED for overall patients. The concept of ED zoning is not a new one in modern emergency medicine. Some EDs have established a fast-track zone [18] or comprehensive older person’s evaluation zone [19] to reduce LOS. Some EDs also have a designated space for surgical interventions, often called an “ED operating room” or “procedure room” [20]. Patients are transferred to the resuscitation bay for physiological monitoring when procedural sedation is required. There is no requirement for a separate group of medical personnel to maintain procedural quality and process control or manage instruments or physiological monitors.

The establishment of the EPZ was an attempt to increase the throughput capacity of the ED by redesigning its physical environment and introducing a dedicated team to handle all emergency procedures, including vital sign and process monitoring. In a systematic review article, James et al. [4] divided the impact of crowding and inefficiencies in the ED into seven major categories: operational metrics, patient outcomes and adverse events, quality measures, access to care, patient experience, educational experience, and financial health. In this study, the establishment of an EPZ addressed these inefficiencies and helped to reduce the impact of ED crowding on the length of stay during the post-EPZ period.

Prior to the establishment of the EPZ in the ED, most clinical procedures were performed at the patient’s bedside, with the exception of an allotted area for stable trauma patients to undergo surgical procedures, such as suturing and cast fixation. However, ED overcrowding remains a major problem in Taiwan [12]. Owing to the lack of space and congestion of emergency beds, not every patient has enough independent space to undergo relevant treatments, which not only limits the operating space but also jeopardizes the patient’s privacy.

Thus, the establishment of an EPZ is beneficial in many ways. First, independent areas and healthcare providers for clinical procedures alleviated delayed examinations and teaching inconveniences. The emergency residents were able to perform a greater number of clinical procedures, including POCUS, CVC, thoracotomy, and lumbar puncture, under the supervision of the attending physician. Second, the relocation of all instruments and consumables to one area facilitated easier access to and management of resources. Third, a separate procedure zone helped maintain patient privacy and safety during monitoring, which is of the utmost importance in emergency medicine. Finally, this separate area could also be utilized for teaching purposes. In conclusion, the establishment of the EPZ improved the efficiency and safety of ED procedures and teaching, benefiting both healthcare providers and patients.

The EPZ area is operated by a separate resident-attending physician-nurse practitioner team. As a result, it is more convenient for the attending physician to conduct a POCUS examination, or any other procedure that is diagnostic in nature, at the patient’s bedside. This provides an additional opportunity to reevaluate the patient’s condition. Furthermore, the ability to perform ultrasound scans and the proficiency of attending residents in performing various procedures also improved significantly. Therefore, future studies should elaborate further on these factors.

Medical order codes were collected to evaluate emergency medical orders before and after the establishment of the EPZ. However, some emergency medical orders were not issued prior to the establishment of the EPZ, making it impossible to assess their implementation status. Therefore, the effectiveness of the resident training was evaluated based only on the number of procedures performed. There is an alternative assessment method to evaluate the training process, such as the number of procedures attempted, the length of each procedure, and the incidence of complications post-procedure. In addition to the EPZ, the patient wait time may also be affected by other factors that cannot be excluded in the present study.

The establishment of an EPZ does not affect resuscitative procedures such as cricothyrotomy, emergency thoracotomy, and Sengstaken–Blakemore tube placement, which are less frequently performed. Owing to limitations in spatial configuration, physicians with diagnostic and procedural skills are divided, which may weaken their overall assessment and point-of-care spirit. This was difficult to quantitatively assess in this study; therefore, it is an issue that requires further consideration. Our study had limitations, as we did not collect data on patient readmission rates post-discharge. This could be addressed in future research, which will also explore the effect of our intervention on board-admitted patients, as our current study only found a small reduction of 8 min in LOS-ED for patients discharged after ultrasound scans or emergency procedures.

ED congestion is multifactorial, requiring consideration of not only throughput but also input and output factors. The benefits of the EPZ beyond LOS-ED reduction include the creation of vacant physician positions, improved patient experience, and an environment that can facilitate teaching. The EPZ model is innovative and experimental, serving as a potential direction for other hospital emergency departments seeking to change their flow process.

## 5. Conclusions

The establishment of a procedure zone in the ED had a significant impact on the efficiency of procedures and the LOS-ED, especially in terms of POCUS. The change in workflow improved the efficiency of diagnosis and disposition, which shortened the length of the hospital stay. The EPZ improved the separated area for clinical procedures, which alleviated delayed examinations and teaching disturbances, as well as provided numerous benefits such as improved management, patient privacy and safety, and teaching opportunities. The unique aspect of the EPZ was the provision of a separate group of medical personnel who maintained procedural quality and process control, and managed instruments or physiological monitors. The emergency zone concept is not new; however, we have provided a novel model for patient flow. Our study concluded that the establishment of an EPZ is an effective way to increase the throughput capacity of an ED by redesigning its physical environment and introducing a dedicated team to handle all emergency procedures, including vital sign and process monitoring. Although the establishment of an EPZ has advantages, the fact that attending physicians and physicians performing the procedures are separated into different teams weakens the spirit of primary care in the ED. This is an important area that requires careful consideration and further improvement.

## Figures and Tables

**Table 1:** Basic characteristics of patients in the emergency department during the pre-emergency procedure zone (EPZ) and post-EPZ periods.

		Pre-EPZ *N* = 137,141 *n* (%)	Post-EPZ *N* = 118,386 *n* (%)	*p* Value
Age	Adults (≥18 years)	113,900 (83.1)	102,539 (86.6)	<0.001
	Children (<18 years)	23,241 (16.9)	15,847 (13.4)	
Gender	Male	68,424 (49.9)	58,583 (49.5)	0.039
	Female	68,717 (50.1)	59,803 (50.5)	
Mechanism	Trauma	41,503 (30.3)	27,984 (23.6)	<0.001
	Non-trauma	95,638 (69.7)	90,402 (76.4)	
Triage acuity level	1	5755 (4.2)	5593 (4.7)	<0.001
	2	20,727 (15.1)	21,020 (17.8)	
	3	103,450 (75.4)	87,902 (74.3)	
	4	6661 (4.9)	2863 (2.4)	
	5	548 (0.4)	1008 (0.9)	
Disposition	Direct discharge from the emergency department	96,814 (70.6)	96,215 (81.3)	<0.001
Mean daily patient volume		249.8 (0.1)	216.8 (0.1)	<0.001

**Table 2:** Procedures that were performed during the pre-EPZ and post-EPZ periods.

		Pre-EPZ *N* = 137,141 *n* (%)	Post-EPZ *N* = 118,386 *n* (%)	*p* Value
Procedure	Suture	6533 (4.76)	4797 (4.05)	<0.001
	CVC insertion	612 (0.45)	1572 (1.33)	<0.001
	Chest tube or pigtail placement	292 (0.21)	348 (0.29)	<0.001
	Thoracentesis	543 (0.40)	524 (0.44)	0.073
	Abdominocentesis	396 (0.29)	281 (0.24)	0.013
	Arthrocentesis	42 (0.03)	129 (0.11)	<0.001
	Lumbar puncture	150 (0.11)	408 (0.34)	<0.001
	Incision and drainage	26 (0.02)	123 (0.10)	<0.001
	Reduction	199 (0.15)	183 (0.15)	0.571
	Splint	378 (0.28)	253 (0.21)	0.002
Ultrasound	Total of ultrasound scans	10,060 (7.34)	12,053 (10.18)	<0.001
	Ultrasound performed in the emergency department (ED)	2026 (1.48)	5370 (4.54)	<0.001
	Focused abdominal sonography for trauma (FAST) in the ED	1426 (1.04)	2704 (2.28)	<0.001
	Non-FAST in the ED	600 (0.44)	2666 (2.25)	<0.001
	Ultrasound performed in the radiology or cardiology department	8034 (5.86)	6683 (5.65)	0.022

**Table 3:** Procedures that were performed for those who were directly discharged during the pre-EPZ and post-EPZ periods.

		Pre-EPZ *N* = 96,814 *n* (%)	Post-EPZ *N* = 96,215 *n* (%)	*p* Value
Procedure	Suture	5507 (5.69)	3938 (4.09)	<0.001
	CVC insertion	18 (0.02)	67 (0.07)	<0.001
	Chest tube or pigtail placement	10 (0.01)	8 (0.01)	0.824
	Thoracentesis	157 (0.16)	153 (0.16)	0.908
	Abdominocentesis	210 (0.22)	146 (0.15)	<0.001
	Arthrocentesis	17 (0.02)	64 (0.07)	<0.001
	Lumbar puncture	33 (0.03)	133 (0.14)	<0.001
	Incision and drainage	12 (0.01)	86 (0.09)	<0.001
	Reduction	157 (0.16)	144 (0.15)	0.523
	Splint	269 (0.28)	189 (0.20)	0.003
Ultrasound	Overall	3814 (3.94)	5487 (5.7)	<0.001
	Ultrasound performed in emergency department (ED)	1257 (1.30)	3339 (3.47)	<0.001
	Focused abdominal sonography for trauma (FAST) in the ED	902 (0.93)	1731 (1.80)	<0.001
	Non-FAST in the ED	355 (0.37)	1608 (1.67)	<0.001
	Ultrasound performed in the radiology or cardiology department	2557 (2.64)	2148 (2.23)	<0.001

**Table 4:** The length of stay in the emergency department (LOS-ED) for patients who were directly discharged from the emergency department during the pre-EPZ and post-EPZ periods.

	Pre-EPZ Median (IQR) (min)	Post-EPZ Median (IQR) (min)	*p* Value
The LOS-ED for all patients	110 (53–127)	102 (33–208)	<0.001
The LOS-ED for patients who underwent procedures			
Suture	72 (43–141)	105 (64–186)	<0.001
CVC	1154 (308–1658)	1198 (476–2076)	0.687
Chest tube or pigtail placement	1425 (236–2684)	288 (213–479)	0.083
Thoracentesis	379 (224.3–1103.5)	481 (285.3–1275.8)	0.018
Abdominocentesis	229 (136–384)	265 (149–410)	0.276
Arthrocentesis	258 (151–662)	241 (154–581)	0.672
Lumbar puncture	1222 (469.3–1636.8)	1271 (720.8–2270.5)	0.304
I&D	333.5 (181–2332.5)	109 (54–227)	0.002
Reduction	107 (62.8–183.3)	150 (80.0–220.0)	0.007
Splint	156 (111.8–231.3)	189 (119.0–272.5)	0.007
The LOS-ED for patients who underwent ultrasound scans			
Overall	365 (166–926)	270 (143–551)	<0.001
Ultrasound performed in the emergency department (ED)	124 (68–280)	180 (109–305)	<0.001
Focused abdominal sonography for trauma (FAST) in the ED	112 (63–238)	158 (93–277.8)	<0.001
Non-FAST in the ED	166 (86–335)	204 (129–339)	<0.001
Ultrasound performed in the radiology or cardiology department	562 (297–1203)	535 (309–1254)	0.470

## Data Availability

Data can be made available on request from established research groups with an appropriate data-sharing agreement. Please contact the corresponding author for data sharing.

## Abbreviations

CVC	central venous catheter
ED	emergency department
EPZ	emergency procedure zone
FAST	focused abdominal sonography for trauma
LOS-ED	length of stay in the emergency department
POCUS	point-of-care ultrasound
